# The High Prevalence of Continuous Paradoxical Breathing During Sleep in Children With Obesity and Its Relationship With Obstructive Sleep Apnea

**DOI:** 10.7759/cureus.77479

**Published:** 2025-01-15

**Authors:** Amal R Al-Naimi, Nadine Asir, Antonisamy Belavendra, Ibrahim Janahi, Ahmed Abushahin, Mutasim Abu-Hasan

**Affiliations:** 1 Pediatric Pulmonology, Sidra Medicine, Doha, QAT; 2 Pediatrics, Sidra Medicine, Doha, QAT

**Keywords:** asynchronous breathing, obesity, obstructive sleep apnea, paradoxical breathing, polysomnography, sleep disorder breathing

## Abstract

Objective: The clinical significance of continuous paradoxical breathing (CPB) during sleep in patients with obesity, and its relationship to upper airway obstruction versus altered chest wall mechanics is not well-studied. We evaluated the prevalence of CPB and its relationship to obstructive sleep apnea (OSA), BMI, sleep position, sleep stage, O_2 _saturation, and sleep-related symptoms in children with obesity.

Methods: All polysomnography (PSG) studies in children with obesity (BMI > 95th percentile) between 2016 and 2022 were evaluated. CPB was defined as complete opposition of chest and abdominal wall signals for at least 50% of rapid eye movement (REM) sleep and/or nonrapid eye movement (NREM) sleep. OSA was defined as an apnea-hypopnea index (AHI) of >1.5 events/hours of sleep. CPB was considered REM-only if present during REM sleep and supine-only if present in the supine position. Multivariate regression analysis was used to evaluate the determinants of CPB.

Results: A total of 196 children (125 males and 71 females) were included. The mean (SD) age was 12.8 (3.4) years. Median (interquartile range (IQR)) BMI was 39 (32.5-46.2) kg/m^2^. OSA was present in 117 patients with a prevalence rate of 59.7%. CPB was identified in 133 patients with a prevalence rate of 67.9%%. CPB was REM-only in 16 (10.9%) patients, and supine-only in 63 (43.2%) patients. There was no correlation between CPB and AHI (p = 0.96), and no correlation was found between CPB and BMI (p = 0.08). There was also no correlation between CPB and O_2_ saturation. However, there was a positive correlation between supine-only CPB and clinical symptoms (p = 0.05).

Conclusion: CPB is highly prevalent in symptomatic obese children and is likely related to chest wall mechanics and not related to OSA.

## Introduction

Childhood obesity is a global epidemic that continues to be on the rise, especially during the post-COVID-19 era. Approximately 340 million children and adolescents have obesity worldwide [1]. Childhood obesity is associated with multiple comorbidities, including systemic hypertension, insulin resistance, systemic inflammation, and sleep-disordered breathing (SDB) [2]. Obstructive sleep apnea (OSA) is the most common SDB in children with obesity. Children with obesity are reported to have a four- to fivefold increased risk of OSA compared to lean controls. Every unit increase in BMI is associated with a 35% increase in the apnea-hypopnea index (AHI) [3-5]. Excess adipose tissue in the neck, pharynx, and tongue base in obese individuals can result in the narrowing of the upper airways and contribute to the high risk of OSA [3,6].

Polysomnography (PSG) is the standard diagnostic test for OSA in both children and adults. AHI, defined as the number of apnea and hypopnea events per hour of sleep, is the main PSG parameter used to diagnose OSA and estimate its severity. Obstructive events are identified during PSG as a significant decrease or complete cessation of airflow with continued or increased respiratory effort. The increase in respiratory effort sometimes manifests as a paradoxical breathing pattern. The presence of this paradoxical breathing is used as one of the criteria that differentiates obstructive from nonobstructive respiratory events [7,8]. Normally, both chest wall and abdominal wall signals are synchronous (i.e., in phase), with the simultaneous rise of both chest wall and abdominal wall during inspiration and expiration. In patients with OSA, the increased intrathoracic pressures needed to overcome upper airway narrowing causes the chest to be depressed and the abdomen to protrude simultaneously during inspiration, and the reverse happens during expiration which leads to the paradoxical breathing pattern.

Continuous paradoxical breathing (CPB) has also been observed in patients with neuromuscular disease which is attributed to the decreased chest wall compliance in these patients [9,10]. CPB has also been reported in healthy infants and young children and is also thought to be related to the relatively increased chest wall compliance in this age group compared to older children.

We have frequently observed CPB during sleep in children with obesity who undergo sleep studies to evaluate for OSA. This abnormal breathing pattern has not been well-studied in this population. The paradoxical breathing pattern can be attributed to the increased propensity for OSA in obese individuals and the associated increase in upper airway resistance which requires large swings in intrathoracic pressures. CPB in obese individuals can also be due to their altered chest wall mechanics. Large swings in intrathoracic pressure are required due to the increased weight on the chest wall and abdomen, which can in turn lead to paradoxical breathing.

The prevalence of CPB in obese subjects is not widely reported. Also, its relationship to OSA versus altered chest wall mechanics has not been well-studied. Such studies have significant implications for future diagnostic and therapeutic approaches to obesity-related SDB. In this study, we aimed to evaluate the prevalence of CPB during sleep in our population of obese children and characterize its relationship to sleep stage, sleep position, OSA, BMI, clinical symptoms, and oxygen saturation.

## Materials and methods

We retrieved diagnostic PSG studies for all children (1-18 years old) with obesity who were referred to the Sidra Medicine Sleep Lab (Doha, Qatar) to evaluate for OSA between September 1, 2016, and August 30, 2022. Demographic and clinical data were collected from patients’ electronic medical records. Obesity was defined as BMI > 95th percentile for age. We excluded patients with comorbid conditions such as neuromuscular diseases; Down syndrome; craniofacial abnormalities; achondroplasia; rapid-onset obesity with hypoventilation, hypothalamic dysfunction, autonomic dysregulation (ROHHAD) syndrome; and Prader-Willi syndrome.

All PSG studies were performed using a standard montage of sensors including electroencephalogram (EEG), electrooculogram (EOG), electromyogram (EMG), nasal pressure, nasal thermistor, chest and abdominal wall motion, limb movement, body position, pulse oximetry, transcutaneous CO_2_, and video recording. Chest wall and abdominal wall signals were obtained using respiratory inductance plethysmography (RIP).

PSG studies were scored by sleep lab technicians according to the American Academy of Sleep Medicine (AASM) scoring criteria [11]. The following PSG parameters were extracted from the generated sleep reports: AHI, rapid eye movement (REM)-AHI, average O_^2^_ saturation, nadir O_2_ saturation, and transcutaneous CO_2_. OSA diagnosis was confirmed if AHI was ≥ 1.5 events/hour. OSA was considered mild if AHI was > 1.5 but < 5, moderate if AHI > 5 but < 10, and severe if AHI was > 10 events/hour.

CPB was evaluated manually by examining chest and abdominal wall signals every two-minute epoch from sleep onset till the end of sleep. CPB was considered present if chest and abdominal wall signals were in complete opposition (i.e., out of phase) for at least 50% of each epoch and more than 50% of all epochs. CPB was evaluated separately during REM and nonrapid eye movement (NREM) sleep as well as in different sleep positions. CPB was considered present during REM sleep if >50% of REM epochs have CPB and was considered present during NREM sleep if >50% of NREM epochs have CPB. CPB was considered REM-only if present during REM sleep but absent during NREM sleep. CPB was considered supine-only if present when the patient is in a supine position but absent when the patient is in decubitus or prone position, regardless of sleep stage.

We estimated the prevalence of CPB, REM-only CPB, and supine-only CPB by dividing patients who fulfilled relevant criteria over the total number of study subjects. We evaluated the relationship between CPB and the following clinical and PSG parameters: age, gender, BMI, AHI, sleep efficiency, mean O_2_ saturation, nadir O_2_ saturation, peak transcutaneous CO_2_, and sleep-related symptoms. The study was approved by the Sidra Medicine Institutional Review Board (IRB # 1970717). Obtaining consent from patients and parents was waived due to the retrospective nature of the study. All patients' data were de-identified.

Statistical analysis

Data were summarized as mean and standard deviation (SD) when normally distributed and as median and interquartile range (IQR) when skewed. Multiple linear regression analysis was used to assess the effect of age, gender, BMI, and sleep-related symptoms on AHI and REM-AHI in the study population. AHI and REM-AHI were log-transformed due to skewness. Logistic regression analysis was also used to assess the effect of age, gender, BMI, sleep-related symptoms, and AHI on CPB, REM-only CPB, and supine-only CPB. A p-value of <0.05 was considered statistically significant. All statistical analyses were performed using STATA SE/18.0 (StataCorp LLC, College Station, Texas, USA).

## Results

A total of 196 patients (125 males and 71 females) met the study criteria. The mean (SD) age was 12.8 (3.4) years. The median (IQR) BMI was 39 kg/m^2^ (32.5-46.2). The median (IQR) BMI centile was 99.98% (99.8-100). A total of 162 (82.7%) patients had a history of sleep-related symptoms: 78 (48.1%) of them reported snoring only, and 84 (51.9%) patients reported both snoring and sleep apnea. Detailed demographic and clinical data are shown in Table 1.

**Table 1:** Summary of clinical characteristics of the study subjects (n = 196) BMI: body mass index; n: number of patients ^a^mean (SD) for normally distributed variables; ^b^median (IQR) for nonnormally distributed variables

Characteristics	Total
Age (years)^a^	12.8 (3.4)
Male, n(%)	125 (63.8%)
Female, n(%)	71 (36.2%)
BMI (kg/m^2^)^b^	39.0 (32.5-46.2)
BMI centile^b^	99.8 (99.8-100)
Symptomatic, n(%)	162 (82.7%)
History of adenotonsillectomy, n(%)	16 (8.2%)
Asthma, n(%)	40 (20.4%)
Fatty liver, n(%)	67 (34.2%)

The median (IQR) AHI was 2.5 (0.8-6.9) events/hour. The median (IQR) REM-AHI was 5.5 (1.8-17.7) events/hour. OSA was confirmed in 117 (59.7%) patients. OSA was mild in 46 (39.3%) patients, moderate in 33 (28.2%) patients, and severe in 38 (32.5%) patients. PSG data are detailed in Table 2.

**Table 2:** Polysomnography data summary (n = 196) REM: rapid eye movement; WASO: wakefulness after sleep onset; AHI: apnea-hypopnea index; OSA: obstructive sleep apnea; ETCO_2_: end-tidal carbon dioxide; n: number of patients ^a^mean (SD) for normally distributed variables; ^b^median (IQR) for nonnormally distributed variables

Polysomnography data	
Sleep efficiency^b^	76.3 (63.4-86.5)
Sleep latency^b^	21.5 (8.5-57.4)
REM latency^b^	117 (75.3-184.5)
WASO^b^	77.6 (38-136.4)
REM%^a^	20.2 (7.4)
AHI^b^	2.5 (0.8-6.9)
Patients with OSA, n (%)	117 (59.7%)
Mild OSA	46 (39.3%)
Moderate OSA	33 (28.2%)
Severe OSA	38 (32.5%)
REM-AHI^b^	5.5 (1.8-17.7)
Snoring events during sleep study, n (%)	144 (73%)
% of total sleep time with snoring^b^	0 (0-1)
Average O_2_ saturation^b^	97 (96-98)
Nadir O_2_ saturation^b^	89 (85-92)
% of total sleep time with SpO_2_<90%^b^	0 (0-0.2)
Peak ETCO_2_^a^	46.7 (5.7)
Hypoventilation, n (%)	3 (3%)
Any paradoxical breathing, n(%)	133 (67.9%)
Only REM related, n(%)	21 (15.8%)
Only positional, n(%)	86 (64.7%)

Multivariate regression analysis showed that male gender, BMI, sleep-related symptoms, and snoring were significant predictors of high AHI (p = 0.002, p < 0.001, p = 0.029, and p = 0.050, respectively) (Table 3).

**Table 3:** Multivariate regression analysis for factors that can affect AHI (n = 196) AHI: apnea-hypopnea index; BMI: body mass index; REM: rapid eye movement Estimates of the effect size of all predictors are presented as odds ratios (95% confidence intervals) of the exponentiated β-coefficients (eβ) using Log AHI as outcome variables. Exponentiated β-values indicate the percentage change in the outcome per unit change in the predictor variable. Models were adjusted for age, gender, BMI, symptomatic, snoring, REM related, position related, and paradoxical

Log AHI	Univariate e ^β ^(95%CI)	p-value	Multivariable e ^β ^(95%CI)	p-value
Age, years	1.03 (0.9, 1.1)	0.349	0.93 (0.9, 1.0)	0.043
Gender (male)	2.43 (1.6, 3.7)	<0.001	2.01 (1.3, 3.1)	0.002
BMI (kg/m^2^)	1.06 (1.0, 1.1)	<0.001	1.05 (1.0, 1.1)	<0.001
Symptomatic (yes)	2.24 (1.3, 3.8)	0.004	2.01 (1.1, 3.8)	0.029
Snoring only (yes)	0.58 (0.4, 0.9)	0.014	0.63 (0.4, 1.0)	0.050
REM related (yes)	1.32 (0.6, 2.8)	0.465	1.55 (0.8, 3.2)	0.225
Position related (yes)	0.87 (0.5, 1.4)	0.557	0.74 (0.4, 1.3)	0.268
Paradoxical (yes)	1.04 (0.7, 1.7)	0.855	1.01 (0.6, 1.8)	0.965

BMI, sleep-related symptoms, and snoring (p = 0.012, p = 0.050, and p = 0.037, respectively) were also significant predictors of REM-AHI (Table 4).

**Table 4:** Multivariate regression analysis for factors that can affect REM-AHI (n = 196) REM: rapid eye movement; AHI: apnea-hypopnea index; BMI: body mass index Estimates of the effect size of all predictors are presented as odds ratios (95% confidence intervals) of the exponentiated β-coefficients (eβ) using log REM-AHI as outcome variables. Exponentiated β-values indicate the percentage change in the outcome per unit change in the predictor variable. Models were adjusted for age, gender, BMI, symptomatic, snoring, REM related, position related, and paradoxical

Log REM-AHI	Univariate e ^β ^(95%CI)	p-value	Multivariable e ^β ^(95%CI)	p-value
Age, years	1.0 (0.9, 1.1)	0.992	0.93 (0.9, 1.0)	0.063
Gender (male)	1.87 (1.2, 2.8)	0.003	1.48 (0.9, 2.3)	0.084
BMI (kg/m^2^)	1.04 (1.0, 1.1)	<0.001	1.03 (1.0, 1.1)	0.012
Symptomatic (yes)	1.72 (1.0, 2.9)	0.042	1.91 (1.0, 3.7)	0.050
Snoring only (yes)	0.54 (0.4, 0.8)	0.002	0.61 (0.4, 0.9)	0.037
REM related (yes)	1.20 (0.6, 2.4)	0.606	1.46 (0.7, 3.0)	0.289
Position related (yes)	0.81 (0.5, 1.3)	0.350	0.73 (0.4, 1.3)	0.257
Paradoxical (yes)	0.88 (0.6, 1.4)	0.570	0.96 (0.5, 1.7)	0.891

CPB was present in 133 (67.9%) patients. CPB was REM-only in 16 (10.9%) patients and supine-only in 63 (43.2%). There was no significant correlation between CPB and AHI and between CPB and REM-AHI (p = 0.97, p = 0.89, respectively). Similarly, there was no significant correlation between REM-only CPB and AHI and between REM-only CPB and REM-AHI. There was also no significant correlation between supine-only CPB and AHI and between supine-only CPB and REM-AHI. There was a trend toward a positive correlation between CPB and BMI but did not reach statistical significance (p = 0.08). There was a significant negative correlation between CPB and age (p = 0.04). There was also a significant positive correlation between supine-only CPB and sleep-related symptoms (p = 0.05) (Table 5).

**Table 5:** Logistic regression analysis of predictors: REM-related continuous paradoxical breathing, continuous positional paradoxical breathing, or any continuous paradoxical breathing CPB: continuous paradoxical breathing; REM: rapid eye movement; BMI: body mass index; AHI: apnea-hypopnea index

Variables	Univariate		Multivariable	
	OR (95% CI)	p-value	OR (95% CI)	p-value
REM related CPB* (n = 146)				
Age (years)	0.99 (0.9, 1.2)	0.96	1.06 (0.8, 1.3)	0.53
Gender (male)	1.08 (0.4, 3.1)	0.89	0.96 (0.3, 2.9)	0.94
BMI (kg/m^2^)	0.98 (0.9, 1.0)	0.40	0.96 (0.9, 1.0)	0.29
AHI	0.98 (0.9, 1.1)	0.69	0.99 (0.9, 1.1)	0.91
Symptomatic (yes)	3.57 (0.4, 28.3)	0.23	4.56 (0.5, 41.3)	0.18
Snoring (yes)	1.36 (0.5, 3.9)	0.56	0.91 (0.3, 2.8)	0.88
Positional CPB (n = 146)				
Age (years)	1.02 (0.9, 1.1)	0.69	1.06 (0.9, 1.2)	0.33
Gender (male)	1.36 (0.7, 2.7)	0.37	1.25 (0.6, 2.5)	0.54
BMI (kg/m^2^)	0.99 (0.9, 1.0)	0.40	0.98 (0.9, 1.0)	0.25
AHI	0.98 (0.9, 1.0)	0.33	0.98 (0.9, 1.0)	0.49
Symptomatic (yes)	2.38 (0.9, 6.1)	0.07	2.89 (1.0, 8.4)	0.05
Snoring (yes)	1.38 (0.7, 2.7)	0.34	0.94 (0.4, 1.9)	0.88
Any CPB (n = 191)				
Age (years)	0.93 (0.8, 1.0)	0.14	0.89 (0.8, 1.0)	0.04
Gender (male)	1.16 (0.6, 2.2)	0.66	1.23 (0.6, 2.4)	0.54
BMI (kg/m^2^)	1.02 (0.9, 1.1)	0.25	1.03 (0.9, 1.1)	0.08
AHI	1.0 (0.9, 1.0)	0.69	0.99 (0.9, 1.0)	0.93
Symptomatic (yes)	2.43 (1.1, 5.2)	0.02	1.76 (0.7, 4.2)	0.20
Snoring (yes)	1.62 (0.8, 3.1)	0.15	1.37 (0.6, 2.9)	0.41

**Figure 1:**
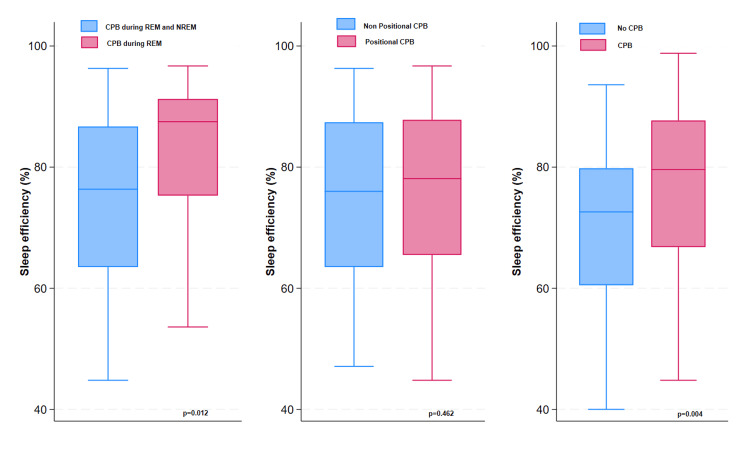
Differences in sleep efficiency between patients with and without REM-related paradoxical breathing, positional paradoxical breathing, or any paradoxical breathing CPB: continuous paradoxical breathing; REM: rapid eye movement

**Figure 2:**
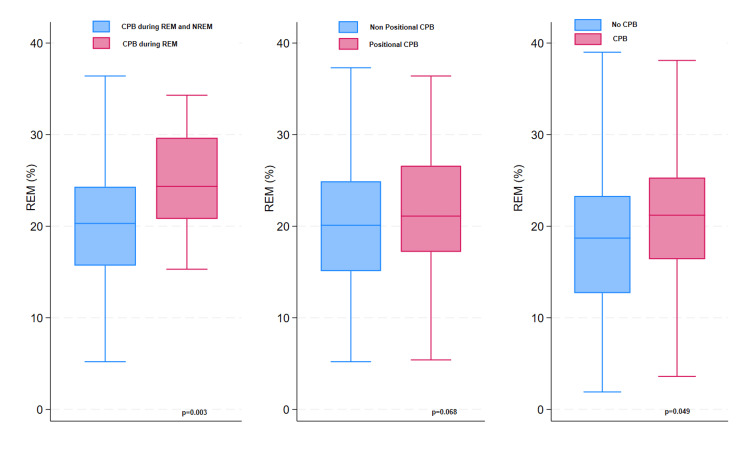
Differences in REM% of total sleep time between patients with and without REM-related continuous paradoxical breathing, positional paradoxical breathing during, or any continuous paradoxical breathing REM: rapid eye movement; CPB: continous paradoxical breathing

There was no significant correlation between CPB and mean O_2 _saturation, nadir O_2 _saturation, or transcutaneous CO_2_ (p = 0.56, p = 0.92, p = 0.81). However, there was a significant positive correlation between CPB and sleep efficiency (p = 0.004). There was also a significant positive correlation between CPB and REM% (p = 0.049) (Figures 1-2). Similarly, there was a significant positive correlation between REM-only CPB and sleep efficiency (p = 0.012) and between REM-only CPB and REM% (p = 0.003) (Figures 1-2).

## Discussion

In this study, we investigated the prevalence of CPB during sleep in obese children and evaluated its relationship to OSA, BMI, sleep stage, sleep position, oxygenation, and clinical symptoms. We found that CPB and OSA were both highly prevalent in obese children (69.6% and 59.7%, respectively). However, we found no strong correlation between CPB and OSA in these children. We also found that only 10.9% of patients had CPB exclusively during REM sleep. These findings suggest that CPB is unlikely to be related to OSA but is more likely to be related to altered chest wall mechanics.

CPB is expected to be more frequent during REM sleep if related to OSA because of the increased upper airway obstruction during REM sleep [12]. The increased frequency of CPB during supine position (69.6%) further indicates that CPB is likely to be related to altered chest wall mechanics and not related to OSA, because of the exclusive effect of the increased weight on thoracic cage mechanics during supine position.

There was a tendency toward a positive correlation between CPB and increased BMI. However, the correlation did not reach statistical significance (p = 0.08). This weak correlation is probably related to the way CPB was defined and quantified in our study. There is also a possibility that variations in the distribution of body fat (not evaluated in our study) affect chest wall compliance differently among patients with obesity. Future studies evaluating the direct relationship between fat distribution, obesity-related changes in lung mechanics and CPB can help improve our understanding of the mechanism by which obesity causes paradoxical breathing.

The paradoxical breathing pattern has been previously reported in healthy infants and young children. These results are consistent with our findings of a significant negative correlation between CPB and age [10,13]. Young children have more compliant chest walls compared to older children which explains the higher likelihood of paradoxical breathing in this age group. Paradoxical breathing patterns have also been widely recognized in patients with neuromuscular diseases due to the decreased chest wall compliance in these patients [9,14].

Paradoxical breathing has also been described in patients with OSA but primarily as one of the criteria used to score obstructive respiratory events. The presence of paradoxical breathing is one of three criteria used to differentiate an obstructive from central events in interpreting PSG studies, besides snoring and flattening of airflow signals [8]. CPB, on the other hand, can potentially occur in patients with OSA due to the increase in upper airway resistance. However, CPB is not part of the diagnostic criteria for upper airway resistance. We found no strong correlation between CPB and OSA in our population which suggests that upper airway resistance is not likely to be the cause of CPB in obesity. Also, the low prevalence of REM-only CPB in obese children and the lack of correlation between REM-only CPB and REM-AHI further confirms the conclusion that CPB is not related to OSA.

Very few studies have previously evaluated the prevalence of CPB during sleep in healthy and nonhealthy populations. Traeger et al. evaluated breathing patterns during sleep in 66 healthy children (age 2-9 years) using either respiratory induction plethysmography (RIP) or a piezoelectric chest belt. Paradoxical breathing was detected more frequently using a piezoelectric chest belt (in 40 ± 24% of epochs) compared to paradoxical breathing detected using the RIP signal (in 1.5 ± 3.3% of epochs) [15]. In our study, we used RIP signals to evaluate CPB. The wide variation in the prevalence of paradoxical breathing due to differences in detection methods highlights the importance of standardizing the techniques used to detect paradoxical breathing in future clinical and research applications.

Unfortunately, there is no standard or conventional definition of CPB. There are also no validated criteria for estimating its severity in children or adults. To define CPB, we arbitrarily used the presence of complete opposition of chest wall vs. abdominal wall movement for more than 50% of each two-minute epoch of sleep with CPB being present in more than 50% of REM epochs or more than 50% of NREM epochs. Our rationale for such a definition was to exclude patients with intermittent or infrequent paradoxical breathing and to include patients with marked CPB patterns for more than half of the sleeping time. A previous pediatric study used an index for asynchronous breathing, called the Labored Breathing Index (LBI), which is defined as the summation of integrals of inspiratory chest and abdomen signals over a summation of tidal volume signal in order to evaluate CPB during sleep in children. Perfect synchrony of chest and abdomen motion resulted in a score of one and any degree of asynchrony resulted in a score of higher than one. Using this definition, the study showed a significant correlation between high LBI and young age. The study also showed a strong correlation between high LBI and SDB [16].

Studies evaluating CPB in obese children or adults are severely lacking. One study by Silvestri et al. reviewed 32 obese children with a mean age of 8.6 (2.7-13.8) years and found that 84% of the patients had a paradoxical inspiratory movement of the chest ≥ 25% of the total sleep time [17]. The difference in prevalence between this study and our study is likely related to differences in CPB definitions. There are no available studies evaluating the relationship between CPB and BMI, OSA, clinical symptoms, sleep position, sleep stage, and other sleep parameters similar to this study.

We found no correlation between CPB and other physiologic sleep parameters including O_2_ saturation and CO_2_. This suggests that CPB is not associated with gas exchange disturbances during sleep. However, this does not exclude other potential deleterious health effects related to CPB on obese individuals. Future studies are needed to evaluate the effects of CPB on other physiological measures during sleep such as intrapleural pressure measurements using esophageal balloon manometry. Also, studies evaluating the effects of CPB on short-term and long-term clinical outcomes are desperately needed.

We found a strong correlation between positional CPB and history of sleep-related symptoms which suggests that paradoxical breathing in obese children is a clinically significant parameter and may require treatment. The positive correlation between CPB and increased sleep efficiency and increased REM% argues against CPB causing major sleep disruptions. Prospective future studies are needed to evaluate the effects of CPB on sleep quality, daytime sleepiness, and cardiovascular and cognitive functions. Such studies will help determine the clinical importance and the need to treat CPB in obese patients. Furthermore, future studies are also needed to evaluate the effect of treating CPB using weight loss programs, sleep positioning, and/or continuous positive airway pressure (CPAP), even in the absence of OSA.

The study is limited by the arbitrary definition used for CPB which may lead to possible overestimation or underestimation of the clinically relevant CBP. In addition, the study is also limited by not evaluating the severity of CPB. Both limitations are due to a lack of established definitions. Future standardization of CPB and its severity is essential.

## Conclusions

In conclusion, abnormal CPB is very prevalent in obese symptomatic children and is more likely to be related to altered chest wall mechanics and is not likely to be related to OSA. Future studies are needed to standardize CPB measurement, evaluate its clinical significance, and determine the need for treatment.
